# The Mechanical Leech of MM. Alexandre & Co. of Paris

**Published:** 1849-09

**Authors:** 


*The Mechanical Leech of MM. Alexandre & Co. of Paris.*—This apparatus consists essentially of two parts —an instrument for puncturing the skin, and another for promoting the flow of blood by removing atmospheric pressure from the punctured part. The puncture is effected by a lancet, the blade of which has the form of the cutting apparatus of the leech. This lancet is fixed in the mouth of a tube, and projects about the eighth of an inch beyond the edge of the tube. It may be elevated by a small lever, so that its point shall be within the tube, in which position it is secured by a catch. Attached to the opposite end of the tube, by a piece of vulcanized India-rubber, which acts as a spring, is a piston, which is pressed down by a rod, and on removing the pressure, is drawn back by the India-rubber spring. The piston being pressed down, the open end of the tube in which the lancet is fixed, is placed over the part to be punctured; the pressure is now removed, when the piston is drawn back by the spring, and exhausting the air within the tube, the skin is forced up into the mouth of the tube. On loosening the lever, by which the lancet has been elevated, the latter is drawn down by a spring, also of vulcanized India-rubber, so as to effect the puncture. The cutting instrument is now removed, and a glass tube with a piston, similar to that already described, is placed over the puncture, the air within being exhausted so that the tube adheres to the part, and the blood flows freely into it. Half a dozen or a dozen tubes, each of which would draw as much blood as a large leech, might be thus attached in two or three minutes. The apparatus, consisting of a cutting instrument and six or twelve suction tubes, together with sundry implements for cleaning the lancet and tubes after use, are contained in a small case. It is very neatly got up, and we understand from those who have used it, is very efficient. The idea, however, is not new; so long ago as the year 1813, the silver medal was awarded at the Society of Arts to Mr. J. Whitford, of St. Bartholomew's Hospital, for the invention of a somewhat similar apparatus for the same purpose. In Mr. Whitford's apparatus the exhaustion was effected by a syringe, which was found to be inconvenient. The use of vulcanized India-rubber springs, attached to the pistons, by which efficient suction tubes are economically formed, is a great improvement in MM. Alexandre & Co.'s apparatus.—*London Med. Journal*, March, from *Pharm. Journal*, February, 1849.

